# Circulating Th17, Th22, and Th1 Cells Are Elevated in the Guillain-Barré Syndrome and Downregulated by IVIg Treatments

**DOI:** 10.1155/2014/740947

**Published:** 2014-05-12

**Authors:** Shujuan Li, Tao Jin, Hong-Liang Zhang, Hong Yu, Fanhua Meng, Hernan Concha Quezada, Jie Zhu

**Affiliations:** ^1^Department of Neurology, the First Hospital, Jilin University, Changchun 130021, China; ^2^Department of Neurology, Wuxi People's Hospital, Nanjing Medical University, Wuxi 214023, China; ^3^Department of Neurobiology, Care Sciences and Society, Karolinska Institute, Karolinska University Hospital, Huddinge, 14186 Stockholm, Sweden; ^4^Department of Otorhinolaryngology, Head and Neck Surgery, the First Hospital, Jilin University, Changchun 130021, China; ^5^Center for Infectious Medicine, Department of Medicine, Karolinska Institute, Karolinska University Hospital, Huddinge, 14186 Stockholm, Sweden

## Abstract

The Guillain-Barré syndrome (GBS) is considered a T helper 1 (Th1) cells-mediated acute inflammatory peripheral neuropathy. However, some changes in GBS could not be explained completely by Th1 cells pathogenic role. Recently, Th17 cells have been identified and can mediate tissue inflammation and autoimmune response. Therefore, a study on the role of Th17 and Th22 cells and their cytokines in GBS is necessary for exploring the pathogenesis of GBS. Here, we detected the frequency of Th1, Th17, and Th22 cells by using 4-color flow cytometry and we detected the plasma levels of IL-17 and IL-22 by ELISA in GBS patients, relapsing-remitting multiple sclerosis patients at the acute phase of relapse, viral encephalitis or meningitis patients and healthy controls. Our data showed that the frequency of circulating Th1, Th17, and Th22 cells was significantly increased in GBS patients. The plasma levels of IL-17 and IL-22 in GBS and relapsing-remitting multiple sclerosis at the acute phase of relapse were also markedly elevated. Enhanced circulating Th22 cells were correlated with GBS severity. Intravenous immunoglobulin therapy downregulated Th17, and Th22 cells and the plasma levels of IL-17 and IL-22 in GBS patients. Th17 and Th22 cells may be involved in the pathogenesis of GBS, and intravenous immunoglobulin mediates therapeutic effects by downregulating these cells and their cytokines.

## 1. Introduction 

The Guillain-Barré syndrome (GBS) is an immune-mediated peripheral neuropathy involving both the myelin sheath and axons. Acute inflammatory demyelinating polyneuropathy (AIDP) and acute motor axonal neuropathy (AMAN) are the most common subtypes of GBS [1–4]. The pathogenesis of GBS remains still enigmatic, but it is largely accepted that both cellular and humoral immune responses are involved in the pathogenesis of GBS [5, 6]. AIDP and its animal model experimental autoimmune neuritis (EAN) have hitherto been classified to Th1 cells-mediated disorders [7–9]. Th1 cells, a subset of CD4^+^T (T helper) cells, are dominant in the inflamed nerves at the acute phase of GBS [8], which could produce IFN-*γ* as a major pathogenic cytokine in GBS, because increased IFN-*γ* was seen in the serum of GBS patients at acute phase [10], and higher immunoreactivity for IFN-*γ* was showed in sural nerves biopsies of GBS patients [11]. However, some changes in GBS and EAN could not be explained by Th1 cell pathogenic role. Most likely, it is complex and involves multiple factors in GBS.

T helper 17 (Th17) cells have been identified as an obvious distinct Th population and a novel Th lineage mediating tissue inflammation and autoimmune response in both animal models and humans [12]. Th17 cells can induce local inflammation in the target organs and help B cells to produce antibodies, two of the hallmarks of GBS pathology. Th17 cells mainly produce IL-17A, which could be promoted by IL-23* in vitro* [13] and* in vivo* [14]. In addition to IL-17A, Th17 cells can secrete IL-17F, IL-21, and IL-22 [15], which induces massive tissue reactions by promoting the recruitment of inflammatory cells [16], while IL-22 shows specific biological characters, such as tissue repairing and wound healing [17].

The increased frequency of Th17 and Th22 cells along with higher levels of IL-17A and IL-22 has been found in multiple inflammatory and autoimmune diseases. IL-17A positive cells were enhanced in the lamina propria and epithelium of inflammatory bowel disease [18]. IL-22 presented high quantities in the blood of the patients with Crohn's disease, and IL-22 mRNA expression was elevated predominantly in mouse colitis model [19, 20]. Th1, Th17, and Th22 cells and IL-22 level were enhanced in psoriasis [21]. Th17 cell frequency was higher in the cerebrospinal fluid of the patients with relapsing-remitting multiple sclerosis (MS) during the relapse phase [22]. Recently, we reported an aggravated clinical course of EAN in IFN-*γ* deficient mice, concomitant with an upregulated level of Th17 cells, indicating a pathogenic role of Th17 in EAN [9]. EAN was attenuated by atorvastatin treatment, a lipid lowering drug with anti-inflammatory properties, and the level of IL-17A was decreased in parallel [23].

However, the role of Th17 and Th22 cells as well as their cytokines in the pathogenesis of GBS is still unclear. Here, we detected the frequency of Th1, Th17, and Th22 cells in the peripheral blood and levels of IL-17 and IL-22 in plasma of GBS patients at the acute and the plateau phases to unravel the mechanisms by which Th17 and Th22 as well as their cytokines may play a pathogenic role in GBS.

## 2. Materials and Methods

### 2.1. Patients and Specimens

We recruited 29 GBS patients fulfilling international diagnostic criteria for GBS or its variants [24], 32 other neurological inflammatory disease controls (ONIDs), including 15 MS patients and 17 encephalitis or meningitis infected by virus (VEM) patients, and 20 healthy controls (HC). All subjects were from the Department of Neurology, the First Hospital, Jilin University, Changchun, China, during June 2010 to August 2012. All GBS patients were classified electrophysiologically as AMAN (*n* = 16) and AIDP (*n* = 13), using motor nerve conduction criteria [1]. Severity of GBS was scored by the use of GBS disability scale scores (GDSs), a widely accepted scoring system to assess the functional status of the patients with GBS [25]. The clinical characteristics of the patients with GBS are shown in Table 1. ONIDs included 15 patients with relapsing-remitting MS (RR-MS) meeting the McDonald criteria [26], who showed mono-/multifocal neurological episode lasting for more than 24 hours (h) at the time of sampling after being neurologically stable for more than 30 days and excluding an acute systemic infection (acute relapse; RR-MS/R). The patients with VEM who exhibited the clinical signs and CSF characteristics according to encephalitis or meningitis and positive virus antibodies in CSF detected by enzyme-linked immunosorbent assays (ELISA) as well as excluding other systemic infections were recruited in our study. These patients also did not receive any immune-modulating drugs or other treatments within 3 months. Twenty age- and sex-matched HC (as compared with GBS subjects) were included in the study.

The pretreatment GBS patients were defined as the patients who did not receive any immune-modulating drugs or other treatments within 3 months, and the posttreatment patients were defined as the patients who received treatments with intravenous immunoglobulin (IVIg) at a dose of 0.4 g/kg body weight per day for 5 days consecutively in the acute phase (1–14 days from onset day). Blood was sampled two times at the acute and the plateau phases (15–32 days from onset day) of GBS and one time for ONIDs before any immune-modulating drugs and other treatments. The present study was approved by the Human Ethics Committee of Jilin Province, China, and informed consent was obtained from all patients and HC.

### 2.2. Preparation of Peripheral Blood Mononuclear Cells and Plasma

Blood samples were taken at 07:00 a.m. each day. Ficoll-Paque (1.077 g/mL, GE Healthcare Bio-Science AB, Uppsala, Sweden) density gradient centrifugation was used to separate peripheral blood mononuclear cells (PBMCs). Mononuclear cells were washed twice in phosphate-buffered saline (PBS), and cell viability measured by Trypan blue exclusion was confirmed to exceed 95%. Plasma samples were also collected and cryopreserved at −80°C for further ELISA performance.

### 2.3. Flow Cytometric Analysis

Four-color flow cytometric technique was used in the analysis of surface phenotypes of PBMCs and cytokines expression. Briefly, mononuclear cells were resuspended at 1 × 10^6^ cells/mL in X-VIVO15 medium (Lonza, Basel, Switzerland) and stimulated with phorbol 12-myristate 13-acetate (PMA) (50 ng/ml; Sigma, St. Louis, MO, USA) and ionomycin (1 *μ*g/mL; Sigma) in the presence of Brefeldin A (10 *μ*g/mL; Sigma) for 4 h. Then, mononuclear cells were fixed and permeabilized with the corresponding buffers (eBioscience, San Diego, CA, USA) and stained for CD4 (Clone: SK3), IFN-*γ* (Clone: B27), IL-17A (Clone: SCPL1362), and IL-22 (Clone: 22URTI) at room temperature (RT) using the following mouse anti-human monoclonal antibodies (mAbs) and corresponding isotype control antibodies (Abs) analyzed by flow cytometry: phycoerythrin- (PE-) conjugated IL-22 (eBioscience), FITC-conjugated IFN-*γ* (BD Bioscience, San Jose, CA, USA), Alexa Fluor 647-conjugated IL-17A (BD Bioscience), and PerCP-conjugated CD4 (BD Bioscience).

Subsequently, cells were fixed in 2% paraformaldehyde PBS and stored at 4°C until flow cytometric analysis by FACSCalibur cytometer using CellQuest software (Becton Dickinson, California, USA). To analyze surface markers in combination with intracellular staining of IFN-*γ*, IL-17A, and IL-22, gate 1 was set up on approximately 2 × 10^4^ lymphocytes of the PBMCs in the FSC/SSC plot, followed by gate 2 on CD4^+^ cells; then, we set gate 3 on CD4^+^ IFN-*γ* ^+^ cells and gate 4 on CD4^+^ IFN-*γ* ^−^ cells; IL-17A and IL-22 markers were subsequently analyzed.

### 2.4. ELISA Measurements of IL-17 and IL-22 in Plasma

The levels of IL-17 and IL-22 in plasma were detected by ELISA according to the manufacturer's instructions. Capture mouse anti-human IL-17 (Clone: Monoclonal Mouse IgG2B Clone number 41809) and IL-22 mAbs (Clone: Monoclonal Mouse IgG1 Clone number 142906), detecting biotinylated antibodies reactive with human IL-17 and IL-22, as well as recombinant human IL-17 and IL-22 (all from R&D systems, Minneapolis, USA) were used in this study. Briefly, 96-well ELISA plates with flat bottom (Greiner Bio-One, Frickenhausen, Germany) were coated with 100 *μ*L IL-17 (6 *μ*g/mL) and IL-22 (6 *μ*g/mL) mAbs, respectively, in carbonate bicarbonate buffer (PH 9.6) and kept at 4°C overnight. After several washes with PBS-Tween 20 (PBST), the wells were blocked with 360 *μ*L per well of 1% bovine serum albumin (BSA) (Sigma) for 60 min at RT. After extensive washing with PBST, 100 *μ*L plasma samples without dilution were added to each well for 2 h of incubation at RT. Thereafter, the plates were washed with PBST and 100 *μ*L biotinylated antibodies IL-17 (0.4 *μ*g/mL) and IL-22 (1 *μ*g/mL), respectively, were added to the wells. After 2 h incubation at RT and three washes with PBST, 100 *μ*L of freshly prepared Streptavidin-HRP (R&D systems) diluted 1 : 200 in PBS with 0.1% BSA was added for 1 h at RT. After three washes with PBST, 100 *μ*L of enzyme substrate which is the ratio of equality combination of stabilized hydrogen peroxide and stabilized tetramethylbenzidine (both from R&D systems) was added to each well. Finally, after 20 min of incubation in the dark, optical density (OD) was determined at 450 nm by enzyme-labeled meter (Bio-Rad 680, Hercules, CA, USA). In order to quantify the plasma levels of IL-17 and IL-22, the standard IL-17 and IL-22 curves were obtained simultaneously by incubating different known concentrations of recombinant IL-17 (0, 1.56, 3.13, 6.25, 12.50, 25, 50, and 100 pg/mL) and IL-22 (0, 31.25, 62.50, 125, 250, 500, 1000, and 2000 pg/mL). OD values measured from the standard concentrations of IL-17 and IL-22 were used to plot standard curves using computer software and then were automatically converted to pg/mL by standard curve. In this assay, background absorbencies (wells without coating mAb) were not obvious and were subtracted from the absorbencies of the specimens. All assays were done in duplicate.

### 2.5. Statistical Analysis

Data were expressed as the mean ± SD. For statistical analysis, the differences of mean values were tested with one-way analysis of variance (ANOVA) for multiple comparisons and Student's* t*-test for two groups, using SPSS software (version 17.0). The Spearman correlation coefficient by rank test was used for correlation analysis between two sets of data. *P* values are two-tailed and are considered statistically significant at *P* < 0.05.

## 3. Results 

### 3.1. Clinical Profiles

The clinical characteristics of GBS are presented in Table 1. Twenty-nine GBS patients had manifested acutely progressive weakness of limbs. A history of antecedent illness was present in 62.1% of the patients (upper respiratory tract infectious symptoms in 20.7%, gastrointestinal tract symptoms in 34.5%, and both symptoms in 6.9%). The characteristics regarding the age and gender of subjects within the four groups are presented in Table 2.

### 3.2. Circulating CD4^+^IFN-*γ* ^+^, Th17 (CD4^+^IL-17A^+^), and CD4^+^IL-22^+^ Cells Are Increased in GBS

**Figure 1:**
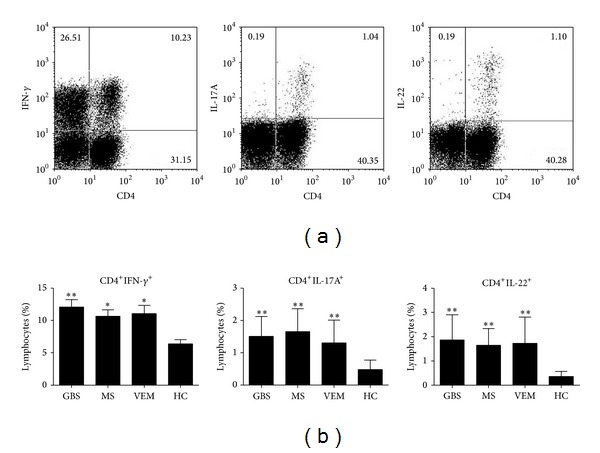
Quantification of circulating CD4^+^IFN-*γ* ^+^, Th17 (CD4^+^IL-17A^+^), and CD4^+^IL-22^+^ cells in each group. (a) Representative two-color dot plot analyses of CD4^+^IFN-*γ* ^+^, CD4^+^IL-17A^+^, and CD4^+^IL-22^+^ cells after stimulation with PMA and ionomycin in the presence of Brefeldin A from a GBS patient. (b) Frequency of CD4^+^IFN-*γ* ^+^, CD4^+^IL-17A^+^, and CD4^+^IL-22^+^ cells on the gated lymphocytes in the FSC/SSC plot in each group: GBS (*n* = 29), RR-MS/R (*n* = 15), VEM (*n* = 17), and HC (*n* = 20). Results are represented as the mean ± SD. Statistical significance was indicated: *P < 0.01 and **P < 0.001.

After gating on lymphocytes, we quantified CD4^+^IFN-*γ* ^+^ cells, CD4^+^IL-17A^+^ cells, and CD4^+^IL-22^+^ cells (Figure 1(a)). When comparing the data of the different groups, we observed that the patients with GBS and ONIDs had a higher frequency of CD4^+^IFN-*γ* ^+^ cells (for GBS, *P* < 0.001; for RR-MS/R, *P* = 0.007; and for VEM, *P* = 0.002, resp.), CD4^+^IL-17A^+^ cells (all comparisons, *P* < 0.001), and CD4^+^IL-22^+^ cells (all comparisons, *P* < 0.001) than HC (Figure 1(b)), while there were no significant differences among GBS and the groups of ONIDs.

### 3.3. Circulating Th1, Th1/Th17, Th17 (CD4^+^IFN-*γ* ^−^IL-17A^+^IL-22^−^, CD4^+^IFN-*γ* ^−^IL-17A^+^IL-22^+^), and Th22 Cells Are Enhanced in GBS

**Figure 2:**
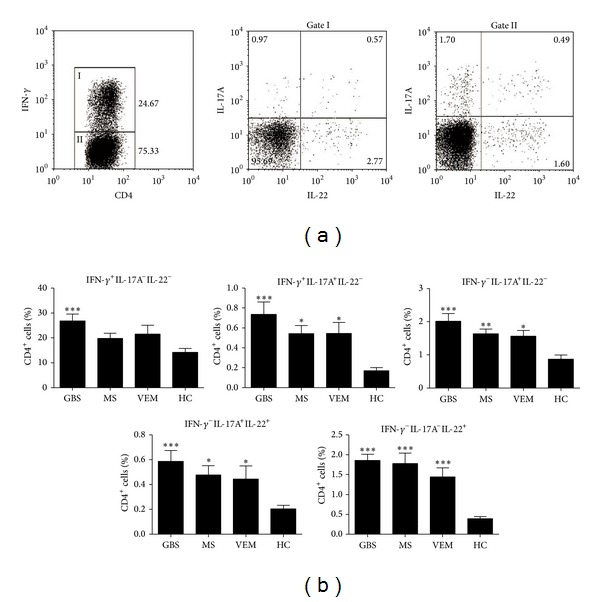
Percentages of circulating Th1 (IFN-*γ* ^+^IL-17A^−^IL-22^−^), Th1/Th17 (IFN-*γ* ^+^IL-17A^+^IL-22^−^), Th17 (IFN-*γ* ^−^IL-17A^+^IL-22^−^, IFN-*γ* ^−^IL-17A^+^IL-22^+^) and Th22 (IFN-*γ* ^−^IL-17A^−^IL-22^+^) cells on CD4^+^ cells of GBS, ONIDs and HC. (a) Representative four-color dot plot analyses of Th1, Th1/Th17 cells on CD4^+^ IFN-*γ* ^+^ (gate I) cells and Th17 (IFN-*γ* ^−^IL-17A^+^IL-22^−^, IFN-*γ* ^−^IL-17A^+^IL-22^+^), Th22 cells on CD4^+^ IFN-*γ* ^−^ (gate II) cells from the same GBS patient in Figure 1(a). (b) The percentages of Th1, Th1/Th17, Th17 (IFN-*γ* ^−^IL-17A^+^IL-22^−^, IFN-*γ* ^−^IL-17A^+^IL-22^+^) and Th22 cells on CD4^+^ cells in each group. Results are represented as the mean ± SD. Statistical significance was indicated: *P < 0.05, **P < 0.01, ***P < 0.001.

After gating on CD4^+^ cells, we detected the percentages of Th1 (IFN-*γ* ^+^IL-17A^−^IL-22^−^), Th1/Th17 (IFN-*γ* ^+^IL-17A^+^IL-22^−^), Th17 (IFN-*γ* ^−^IL-17A^+^IL-22^−^, IFN-*γ* ^−^IL-17A^+^IL-22^+^), and Th22 (IFN-*γ* ^−^IL-17A^−^IL-22^+^) cells (Figure 2(a)). When we compared the data of circulating cells in the different groups, we found that the patients with GBS had a higher frequency of Th1 cells than HC (*P* < 0.001). There were no significant differences among RR-MS/R, VEM, and HC (Figure 2(b)).

Both GBS and ONIDs had a higher frequency of Th1/Th17 cells than HC (for GBS, *P* < 0.001; for RR-MS/R, *P* = 0.013; and for VEM, *P* = 0.010, resp.). When we compared other cell types among GBS, ONIDs, and HC, the data are as follows: IFN-*γ* ^−^IL-17A^+^IL-22^−^ cells compared with HC (for GBS, *P* < 0.001; for RR-MS/R, *P* = 0.006; and for VEM, *P* = 0.010); IFN-*γ* ^−^IL-17A^+^IL-22^+^ (*P* < 0.001, *P* = 0.018, and *P* = 0.031); Th22 cells (all comparisons, *P* < 0.001). No significant differences of the frequency of these cells were found among GBS and ONIDs (Figure 2(b)).

### 3.4. Elevated Circulating Th22 Cells Are Correlated with GBS Severity, but Not with GBS Subtypes

**Figure 3:**
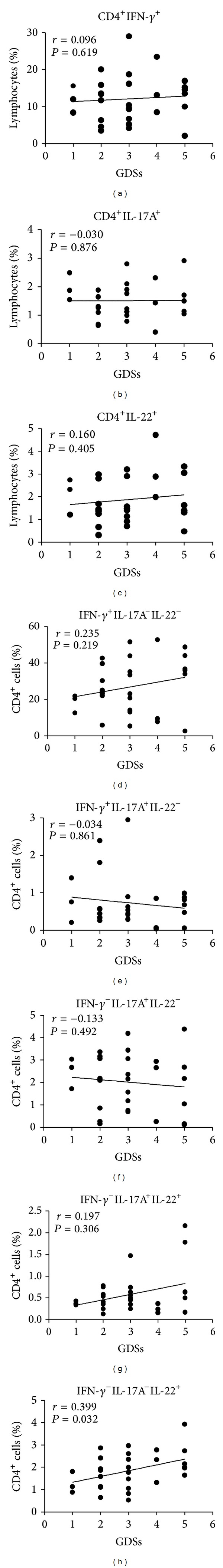
The correlations of circulating CD4^+^IFN-*γ* ^+^, CD4^+^IL-22^+^, Th1 (IFN-*γ* ^+^IL-17A^−^IL-22^−^), Th1/Th17 (IFN-*γ* ^+^IL-17A^+^IL-22^−^), Th17 (CD4^+^IL-17A^+^, IFN-*γ* ^−^IL-17A^+^IL-22^−^, and IFN-*γ* ^−^IL-17A^+^IL-22^+^), and Th22 (IFN-*γ* ^−^IL-17A^−^IL-22^+^) cells on CD4^+^ cells with GBS disability scale scores (GDSs). Dot plot of Th22 cells and GDSs of GBS patients illustrated their correlation (*P* < 0.05) (h). Dot plot of CD4^+^IL-22^+^ cells had an elevated tendency with higher GDSs (c). The Spearman correlation coefficient was used to differentiate between two sets. Each circle = single individual; lines = linear approximation.

To determine the correlation between the elevated cells and GBS severity, we analyzed our data with GDSs by using Spearman's correlation coefficient. Our data revealed that Th22 cells were correlated with GDSs (*r* = 0.399, *P* = 0.032) (Figure 3(h)), though there was no quantitative uniqueness for the frequency of CD4^+^IL-22^+^ cells with GBS severity; the elevated CD4^+^IL-22^+^ cells had a tendency for disease severity status in GBS (*r* = 0.160, *P* = 0.405) (Figure 3(c)); no other cells were correlated with GDSs (Figures 3(a), 3(b), and 3(d)–3(g)). We also compared the frequency of these elevated cells with the GBS subtypes, including AIDP (*n* = 13) and AMAN (*n* = 16), but there was no significant difference between the two subtypes (data not shown).

### 3.5. Higher Plasma Levels of IL-17 and IL-22 in GBS

**Figure 4:**
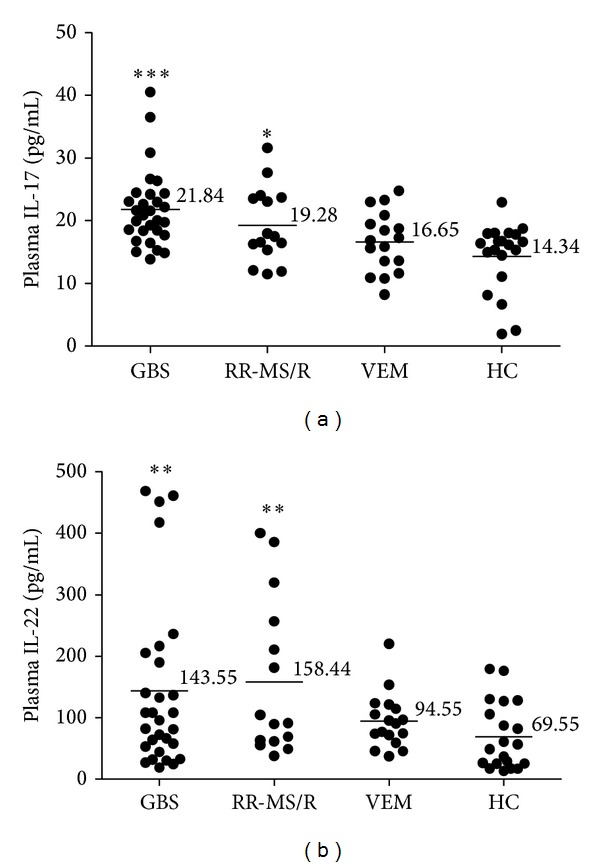
Plasma levels of IL-17 (a) and IL-22 (b) are elevated in GBS patients. Each circle = single individual; horizontal bars = mean. *P < 0.05, **P < 0.01, and ***P < 0.001.

Plasma levels of IL-17 and IL-22 in all groups were also measured by ELISA. Our data demonstrated that plasma level of IL-17 was significantly elevated in GBS and RR-MS/R compared with HC (*P* < 0.001 and *P* = 0.01, resp.). Meanwhile, the IL-22 level was higher in GBS and RR-MS/R than in HC (*P* = 0.009 and *P* = 0.007, resp.). However, there was no significant difference in IL-17 and IL-22 levels between VEM and HC (data not shown) (Figures 4(a) and 4(b)).

### 3.6. IVIg Reduces Inflammatory Cells as well as Plasma Levels of IL-17 and IL-22 in GBS

**Figure 5:**
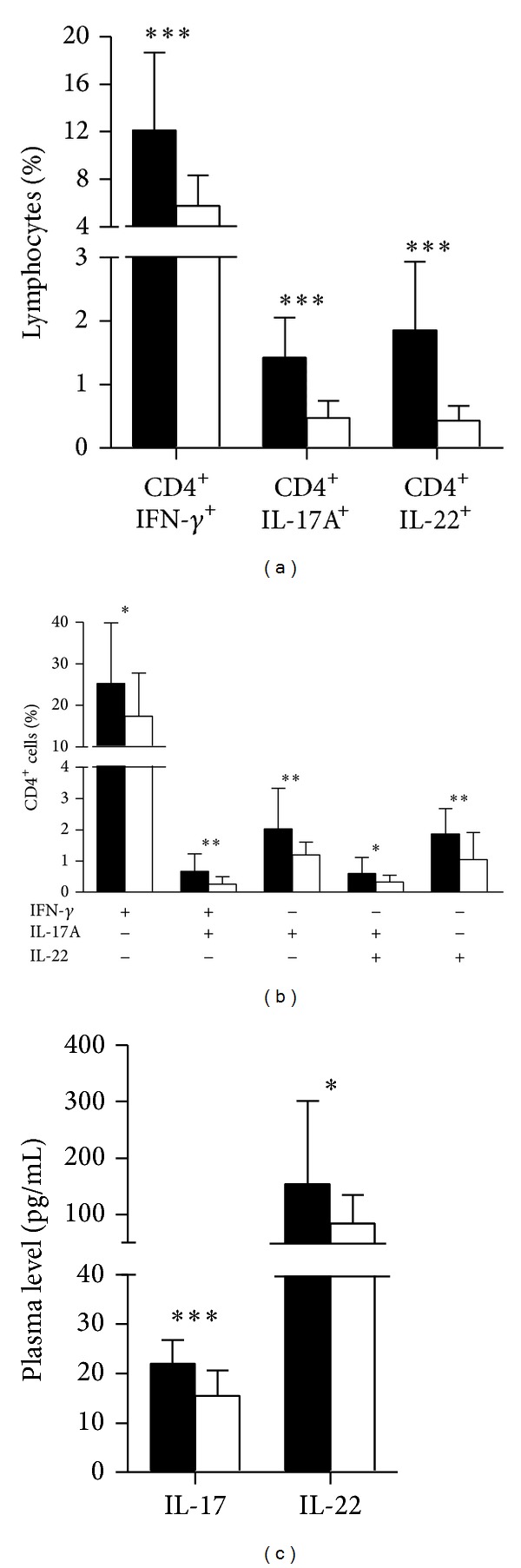
IVIg treatments downregulate CD4^+^IFN-*γ* ^+^, CD4^+^IL-22^+^, and Th1, Th1/Th17, Th17, and Th22 cells and their cytokines at the plateau phase of GBS. A total of 24 GBS patients were treated with IVIg, and Th1, Th17, and Th22 cells as well as plasma levels of IL-17 and IL-22 were determined before (black bar) and after treatment (white bar). (a) Bar plot of the percentages of CD4^+^IFN-*γ* ^+^, Th17 (CD4^+^IL-17A^+^), and CD4^+^IL-22^+^ cells on the gated lymphocytes in the FSC/SSC plot. (b) Bar plot of the percentages of Th1 (IFN-*γ* ^+^IL-17A^−^IL-22^−^), Th1/Th17 (IFN-*γ* ^+^IL-17A^+^IL-22^−^), Th17 (IFN-*γ* ^−^IL-17A^+^IL-22^−^, IFN-*γ* ^−^IL-17A^+^IL-22^+^), and Th22 (IFN-*γ* ^−^IL-17A^−^IL-22^+^) cells on CD4^+^ cells. (c) Bar plot of the levels of plasma of IL-17 and IL-22. Results are represented as the mean ± SD. Statistical significance was indicated: *P < 0.05, **P < 0.01, and ***P < 0.001.

To further understand the effect of IVIg treatment on the inflammatory cells and cytokines, we detected the frequency of these cells by flow cytometry and plasma levels of cytokines by ELISA before and after treatment with IVIg in GBS patients (*n* = 24), who were suffering from more serious clinical signs. Our data showed that the frequency of CD4^+^IFN-*γ* ^+^ cells, CD4^+^IL-17A^+^ cells, and CD4^+^IL-22^+^ cells was more downregulated after IVIg treatment than before treatment (all comparisons, *P* < 0.001) (Figure 5(a)). The similar results were found in Th1 (*P* = 0.035), Th1/Th17 (*P* = 0.006), IFN-*γ* ^−^IL-17A^+^IL-22^−^ (*P* = 0.004), IFN-*γ* ^−^IL-17A^+^IL-22^+^ (*P* = 0.014), and Th22 cells (*P* = 0.001) (Figure 5(b)). Clearly, IVIg treatments also declined the levels of IL-17 and IL-22 in plasma after treatment compared with before treatment (for IL-17, *P* < 0.001; for IL-22, *P* = 0.033) (Figure 5(c)). However, there was no significant difference regarding the levels of these cells and cytokines in GBS patients with (24 cases) and without IVIg treatments (5 cases with slight clinical signs) (data not shown).

## 4. Discussion 

In the present study, our results showed that circulating Th1, Th17, and Th22 cells as well as the levels of IL-17 and IL-22 in plasma were obviously elevated in GBS at the acute phase and IVIg treatments could downregulate these cells and their cytokines at the plateau phase of GBS. Thus, it is speculated that Th17 and Th22 cells as well as IL-17/IL-22 are involved in the initiation and development of GBS and IVIg treatments effectively reduce their levels and attenuateclinical signs of GBS.

Th1 and its cytokines are the pathogenic molecules in GBS as we reported previously [7, 8]. In the present study, we found that Th17 and Th22 cells and their cytokines may also contribute to the pathogenesis of GBS. Our data showed that, among all Th17 subgroups, the CD4^+^IFN-*γ* ^−^IL-17A^+^IL-22^+^ cell population is a relatively small portion compared to CD4^+^IL-17A^+^ and CD4^+^IFN-*γ* ^−^IL-17A^+^IL-22^−^ subgroups. Th17 cell lineage produces not only IL-17A but also IL-22, both of which contribute to the controlling of extracellular bacterial infection by the induction of a powerful immune response [27]. Although both Th17 and Th22 cells can produce IL-22, they are different T helper cell lineages. Th17 cells play an important role in host defense against infections and in tissue inflammation during autoimmunity [28], while Th22 cells are important in epithelial cell homeostasis, as well as in tissue repair and wound healing [17]. IFN-*γ* has an inhibitory effect on the production of IL-17 [29, 30]; conversely, in similar conditions, Th22 cells mainly coproduce IFN-*γ* rather than IL-17 [31].

We delineated several potential mechanisms by which Th17 and Th22 cells and IL-17/IL-22 could participate in the pathogenesis of GBS. Firstly, higher levels of them showed an apparent enhancement of T cell responses in the acute phase of GBS. Secondly, the quantity of Th22 cells had a positive correlation with disease severity of GBS, which suggested that Th22 may play an important role in the development of GBS. In previous studies, Th22 cells and IL-22 often showed to correlate with disease severity, such as in the patients with rheumatoid arthritis [32] and psoriasis [21]. Th17 and Th22 cells of GBS patients at acute phase could express an appropriate cytokine profile, like IL-17, IL-22, and others (IL-6 and TNF-*α*), which can enhance the inflammatory and autoimmune response and conduce to the development of GBS [33, 34]. Finally, increased IL-17 levels in plasma of GBS patients are enhanced homing of inflammatory cells to the peripheral nervous system, which might contribute to the pathogenesis of GBS.

However, in our study, increased Th1, Th1/Th17, Th17, and Th22 cells were also found in ONIDs, and there were no significant differences among GBS and the groups of ONIDs, which indicated that, although the increased cells are associated with GBS, they are also related to other inflammatory and autoimmune disorders; therefore, the increased cells in GBS are not specific for GBS. Similarly, increased inflammatory cytokines and other molecules can be seen in both GBS and ONIDs. It is speculated that Th17 and Th22 cells are not unique pathogenic cells and there could be an intricate network of inflammatory molecules in the pathogenesis of GBS.

High-dose IVIg therapy is an effective treatment in GBS; however, its anti-inflammatory mechanisms remain elusive. It has been reported that IVIg can block the mononuclear phagocytic system, neutralize autoantibodies by anti-idiotype antibodies, interrupt the complement activation cascade, and influence the effect of Fc receptor-mediated activity of immunologically relevant cells [35, 36]. IVIg can inhibit T cell proliferation and its cytokine production [37, 38]. It also can inactivate, silence, or bring about programmed T cell death [39]. Recent studies showed that IVIg inhibits the differentiation and amplification of Th17 cells and the production of their cytokines IL-17A, IL-17F, IL-21, and CCL20 [40]. Moreover, the effect of IVIg is more prominent on the inhibition of IL-17 production from Th17 cells [41]. Unfortunately, so far, the effect of IVIg treatment on Th22 cells differentiation and function has not been explored. However, Sugita et al. reported that, in the presence of anti-TNF-*α* and anti-IL-6 antibodies, Behçet's disease Th22-type T cells failed to produce IL-22 [42]. Thus, it is speculated that IVIg treatment with anti-TNF-*α* property could reduce or inhibit Th22 function probably, which needs to be explored further. In the present study, we firstly reported that IVIg downregulated Th17 and Th22 cells and their effector cytokines IL-17 and IL-22 in GBS patients.

## 5. Conclusions

In summary, elevated circulating Th17 and Th22 cells may contribute to the pathogenesis of GBS. IVIg mediates its therapeutic effects by downregulating these cells and their cytokines in GBS. Our data suggest that antagonists of Th17 and Th22 cells and their cytokines may have therapeutic potentials for alleviating GBS in humans.

## Figures and Tables

**Table 1:** Clinical survey of the patients with GBS.

Number	Sex	Age (years)	Previous infections	Electrophysiologic findings	GDSs at acute/plateau phases	IVIg
1	Female	51	None	Axonal	3/3	No
2	Male	61	None	Demy.	4/3	Yes
3	Male	39	None	Axonal	5/4	Yes
4	Female	19	None	Demy.	5/4	Yes
5	Female	40	URTI	Demy.	2/2	Yes
6	Male	37	GI + URTI	Axonal	3/3	No
7	Male	43	None	Demy.	3/2	Yes
8	Female	17	None	Axonal	5/3	Yes
9	Female	48	URTI	Demy.	2/2	Yes
10	Male	46	URTI	Axonal	2/1	No
11	Male	48	GI	Axonal	2/2	Yes
12	Male	46	None	Demy.	3/3	Yes
13	Female	51	URTI	Demy.	2/1	Yes
14	Male	28	GI	Demy.	3/3	Yes
15	Male	37	GI	Axonal	2/2	Yes
16	Male	18	GI	Axonal	5/3	Yes
17	Male	35	GI	Demy.	3/2	Yes
18	Male	53	URTI	Demy.	4/3	Yes
19	Female	63	URTI	Demy.	3/1	Yes
20	Male	40	GI + URTI	Demy.	4/2	Yes
21	Male	27	GI	Axonal	5/3	Yes
22	Male	49	None	Axonal	2/2	Yes
23	Female	61	None	Axonal	1/1	Yes
24	Male	33	GI	Axonal	1/0	Yes
25	Male	44	GI	Demy.	5/3	Yes
26	Male	46	None	Axonal	3/3	No
27	Male	54	None	Axonal	1/1	No
28	Male	38	GI	Axonal	2/0	Yes
29	Female	29	GI	Axonal	3/1	Yes

GI: gastrointestinal infections; URTI: upper respiratory tract infections; demy.: primary demyelination; axonal: primary axonal injury; IVIg: intravenous immunoglobulin treatments.

**Table 2:** Demographics of subjects in four groups evaluated in this study.

	Male	Female	Age (mean ± SD) (years)
GBS	20	9	41.41 ± 12.37
RR-MS/R	2	13	38.27 ± 12.68
VEM	11	6	34.12 ± 12.70
HC	14	6	40.35 ± 11.71
